# MAP kinase pathways and calcitonin influence CD44 alternate isoform expression in prostate cancer cells

**DOI:** 10.1186/1471-2407-8-260

**Published:** 2008-09-15

**Authors:** Eric W Robbins, Emily A Travanty, Kui Yang, Kenneth A Iczkowski

**Affiliations:** 1 From the Department of Pathology, Anschutz Medical Campus, University of Colorado Denver, Aurora, Colorado, USA

## Abstract

**Background:**

Dysregulated expression and splicing of cell adhesion marker CD44 is found in many types of cancer. In prostate cancer (PC) specifically, the standard isoform (CD44s) has been found to be downregulated compared with benign tissue whereas predominant variant isoform CD44v7-10 is upregulated. Mitogen-activated protein kinase pathways and paracrine calcitonin are two common factors linked to dysregulated expression and splicing of CD44 in cancer. Calcitonin has been found to increase proliferation and invasion in PC acting through the protein kinase A pathway.

**Methods:**

In androgen-independent PC with known high CD44v7-10 expression, CD44 total and CD44v7-10 RNA or protein were assessed in response to exogenous and endogenous calcitonin and to inhibitors of protein kinase A, MEK, JNK, or p38 kinase. Benign cells and calcitonin receptor-negative PC cells were also tested.

**Results:**

MEK or p38 but not JNK reduced CD44 total RNA by 40%–65% in cancer and benign cells. Inhibition of protein kinase A reduced CD44 total and v7-10 protein expression. In calcitonin receptor-positive cells only, calcitonin increased CD44 variant RNA and protein by 3 h and persisting to 48 h, apparently dependent on an uninhibited p38 pathway. Cells with constitutive CT expression showed an increase in CD44v7-10 mRNA but a decrease in CD44 total RNA.

**Conclusion:**

The MEK pathway increases CD44 RNA, while calcitonin, acting through the protein kinase A and p38 pathway, facilitates variant splicing. These findings could be used in the formulation of therapeutic methods for PC targeting CD44 alternate splicing.

## Background

CD44, a transmembrane glycoprotein, is the product of a gene that can undergo extensive alternate splicing. The standard (**CD44s**) isoform is ubiquitous but tissue-specific isoforms may include an assortment of 10 variant (**v**) exons (**CD44v**). CD44 facilitates multiple cellular functions. CD44 enables cell-cell and cell-matrix adhesion – primarily to its main ligand hyaluronan, and links the cell membrane to the actin cytoskeleton, modulating motility. CD44 is universally dysregulated in human cancer, and this imbalance of isoforms allows tumor growth and invasion [1-8]. CD44v are expressed in prostatic secrectory cells while CD44s is found in the whole epithelium. About 30% of cases of prostate cancer (PC) undergo a transition from quiescent to aggressive. Altered CD44 and other adhesion molecules permit this transition in which tumor cells detach, interact with proteins that digest stromal matrix, migrate through matrix, and intravasate into lymphovascular channels.

By isolating RNA from clinical PC specimens, we discovered that the major variant isoform expressed in PC is CD44v7-10. This PC signature was consistently present in both primary and metastatic PC [1-3]. Interference against this CD44v caused a 69% reduction in invasion index compared to untreated control cells[3]. Moreover, PC loses the splicing ability to produce the CD44s expressed in benign prostate[3,9,10]. CD44 must oligomerize to bind matrix ligands or to cause metastasis[11] and variant isoforms, with longer extracellular tails, have altered ability to complex[12]. We found that the CD44v7-10 isoform makes PC cells preferentially bind to fibronectin rather than hyaluronan; re-expression of CD44s causes the predominant ligand to revert from fibronectin back to hyaluronan[4]. In mouse xenografts of PC-3 prostate tumor, forced expression of CD44s reduced growth *in vitro *and tumorigenicity[5], and our use of RNAi against CD44v7-10 in xenografts yielded similar effects (unpublished results).

In PC, calcitonin (CT) acts as a paracrine growth factor that up-regulates CD44 variant[4,6]. In histologic specimens PC, but not benign secretory epithelium, contains CT[13] and its receptor (CTR)[14], and CT exerts paracrine effects that promote proliferation[15], invasion[16], and metastasis[17]. CTR, essential for prostate cancer tumorigenicity[18], is coupled to the transduction protein G_s_α. We have shown that CT promotes alternate splicing leading to CD44v7-10 mRNA and protein[4,6] by acting through G_s_α signaling[3]. G_s_α stimulates the cyclic AMP signaling cascade[17,19] and protein kinase A (PKA)[16].

PKA, in turn, acts on the 3 main MAPK pathways: a growth factor-responsive pathway that uses MAP2K (also called MEK) as key downstream effector; and two stress-activated pathways, c-jun N-terminal kinase (JNK), and p38 kinase, that respond to stress including cytokines, osmotic shock, and irradiation. CD44 variants activate MAPK pathways[20], sometimes by functioning as co-receptors for growth factors[21]. MAPK pathways, in turn, can cause CD44 alternative splicing to include variant exons[22]. Oncogenes such as ras[7,23] and mitogens using the MEK-ERK MAP kinase (MAPK) pathway[7], but not the p38 pathway[24], induce CD44 promoter activity and increase expression of certain CD44v. To test whether these influences modulate RNA levels and alternative splicing of CD44 in PC, we studied the CT signaling system, PKA, and MAPK pathways. CD44 mRNA and protein levels were measured.

## Methods

### Cell lines

PC-3 cells (American Type Culture Collection, Manassas, VA) were incubated in F12-K medium, 10% fetal calf serum, and antibiotics at 37°C in a 5% CO_2 _incubator. G_s_α-QL cells, CT+, CT-, and CTR-cells were gifts of Dr. Girish Shah, Univ. of Louisiana-Monroe[17]. The G_s_α-QL cells were derived from metastasizing PC-3M cells stably transfected by a plasmid that directs expression of mutant, constitutively active G_s_α [17,19]. These three cell lines were grown in RPMI 1640 with L-glutamine, 5% fetal calf serum, 15% horse serum and antibiotics. Benign BPH-1 cells (from Dr. Simon Hayward, Vanderbilt Univ., Nashville, TN) were grown in RPMI with 10% fetal calf serum and antibiotics. For each experiment, cells in a flask were trypsinized and saline washed to remove trypsin. 200,000 cells were plated per well on a 6-well plate. Cells were adherent and 80% confluent for all experiments. Each treatment was applied to three wells, with three other wells as mock treated controls.

### Effect of exogenous calcitonin

PC-3M cells express both CT and CT-receptor (CTR)[16]. To test the effect of solely exogenous CT on CD44, we used the derivative, CT-minus (CT-). CT-cells have endogenous CT stably knocked down to undetectable levels using anti-CT hammerhead ribozymes[25]. Salmon CT (BAChem, Torrance, CA) was used at a physiologic 50 nM dose[14,16], which effectively alters CD44[6], or at 250 nM. The K_d _of CTR is 4–21 nM[15]. To detect acute versus long-acting effects on RNA and protein levels, cells were treated with 50 nM CT and harvested after 3 h or 48 h. To determine whether CT effects on CD44 proteins resulted from *de novo *protein synthesis versus protein stabilization, cycloheximide (10 nM) was given to CT-cells after 3 hr of CT exposure, and cells harvested 1 hr, 3 hr, 6 hr, and 9 hr subsequently, similar to a prior CD44 study[26]. Also tested were highly invasive, CT positive G_s_α-QL cells[17]. Finally, to rule out non-CTR-mediated CT effects, two negative controls were tested: PC-3 cells (shown to be negative for CT receptor[14]), and cells called CTR-, derived from PC-3M cells after anti-CT receptor ribozyme knockdown of CTR[18]. CTR-cells have very low levels of CD44v protein[4].

### Effect of endogenous calcitonin

To test the effect of endogenous CT on CD44 RNA, we used PC-3M cells expressing CT-pcDNA 3.1 plasmid (CT+), which constitutively express CT[25]. 125,000 PC-3M or CT+ cells per well were plated and allowed to grow for 72 hours.

### Inhibition of protein kinase A and MAPK components

G_s_α-QL cells were chosen for these studies because they have the highest baseline CD44v[3]. Protein kinase A inhibitor H-89 (in 50% ethanol) (Calbiochem, La Jolla, CA) was added to cells in fresh medium (1 mL/well) at 1 μM, previously shown effective in nerve cells[23] or at a 10 μM dose. Cells were incubated with H-89 for 24 hours[27] then harvested.

In similar assays, either 10 μl of 1 mM JNK inhibitor (SP600125, Calbiochem) or 12.5 μl of 2 mM MEK inhibitor (PD98059, Calbiochem) in water were added, yielding concentrations previously shown effective: 10 μM for JNK[28] and 25 μM for MEK (personal communication, Dr. Bolin Liu). p38 kinase inhibitor (SB203580, Calbiochem) was used at 10 μM[29,30] in DMSO, and control cells received DMSO only. Cells were incubated for the optimum time of 48 h[28] to show effects.

### Interaction of CT with MAPK pathways

Based on results above, we tested the effect of pretreatment with MEK or p38 kinase inhibitors on CT-mediated alteration of CD44 expression. 25 μM MEK inhibitor or 10 μM p38 kinase inhibitor was added to CT-cells 4 hours prior to administering 50 nM CT. Cells were harvested after 48 h as above.

### Real time TaqMan RNA analysis

Total RNA was prepared from cell pellets using Trizol (Invitrogen, Carlsbad, CA) as described by the manufacturer. RNA was further purified by isopropanol precipitation, resuspended in RNAse-free water, and its concentration measured. Complementary DNA (cDNA) was synthesized from 4 μg total RNA in 20 μl reaction mixture as we did previously[9]. At least triplicate samples were run using a primer/probe set for all CD44v that brackets the entire variant region[6], one for CD44 total that binds a standard exon, and 18S ribosomal RNA. Quantitative PCR reactions were optimized to 4 μg cDNA (0.16 μg with 18S) plus the manufacturer's master mix and primer/probe sets (Applied Biosystems, Foster City, CA) in a volume of 20 μl. The amplification protocol was as follows: hold 50°C 2 min, 95°C 10 min, then 40 cycles of (95°C for 0:15 and 60°C for 1:00) using the ABI Prism 7700 cycler (Perkin-Elmer, Waltham, MA). Primer/probe sets for CD44v were: forward, AACGCTTCAGCCTACTGCAAA; reverse, TCTTCCAAGCCTTCATGTGATG; probe, GATTTGGACAGGACAGGACCTCTTTCAATG. For CD44 total we used forward, CAACTCCATCTGTGCAGCAAA; reverse, GTAACCTCCTGAAGTGCTGCTC; probe, CATATTGCTTCAATGCTTCAGCTCCACCTG. Primer and probe sets for 18S were proprietary to the manufacturer.

### Western blot analysis

Cultured cells were directly lysed in their wells using RIPA buffer (Upstate Biologicals, Lake Placid, NY) with protease inhibitor Complete-mini tablet (Applied Science, Indianapolis, IN). Protein concentration of the cell lysate was estimated by Bradford method. Samples were resolved on SDS-PAGE using 25 μg sample/lane with the NuPAGE system (Invitrogen, Carlsbad, CA). 5 μl of Rainbow protein marker (RPN 756, Amersham, Piscataway, NJ) was run in at least one lane. After electrophoresis for 2 hr, the protein was transferred to PVDF. Three primary antibodies were used. To assess CD44v9 (the largest component of the overexpressed CD44v7-10) the membrane was reacted with neat supernatant from the hybridoma cell line HB-258 (ATCC). CD44 standard was assessed using anti-HCAM (DF1485, Santa Cruz Biologicals, Santa Cruz, CA, 1:2000), which binds all CD44 isoforms. Anti-β-actin antibody (Sigma, St. Louis) was used at a dilution of 1:10,000. Membranes were washed 3 × 15 min in TBS with 20 mM Tris pH 7.5 and 1:1000 dilution of goat anti-mouse IgG antibody labeled with biotin (Bio-Rad) was added at 1:9000 dilution in 5% skim milk for 1 hr. Reactivity was detected using a chemiluminescent system (SuperSignal West Pico Substrate, Pierce Biotechnology, Rockford, IL). Each experimental run was conducted at least twice.

### Statistical analysis

TaqMan data were analyzed by the 2(-ΔΔC_T_) method [31] to determine fold change in gene expression (mock treated cells = 1.00). The ΔC_T _was taken as the difference between the CD44v or CD44 total and the 18S ribosomal RNA C_T_s. The ΔΔC_T _was obtained using the mean ΔC_T _of mock treated cells as calibrator. Each TaqMan result was compared to 1.00 using 2-tailed paired t-test. Statistical significance was set at p < 0.05.

## Results

### Calcitonin increases CD44v

**Figure 1:**
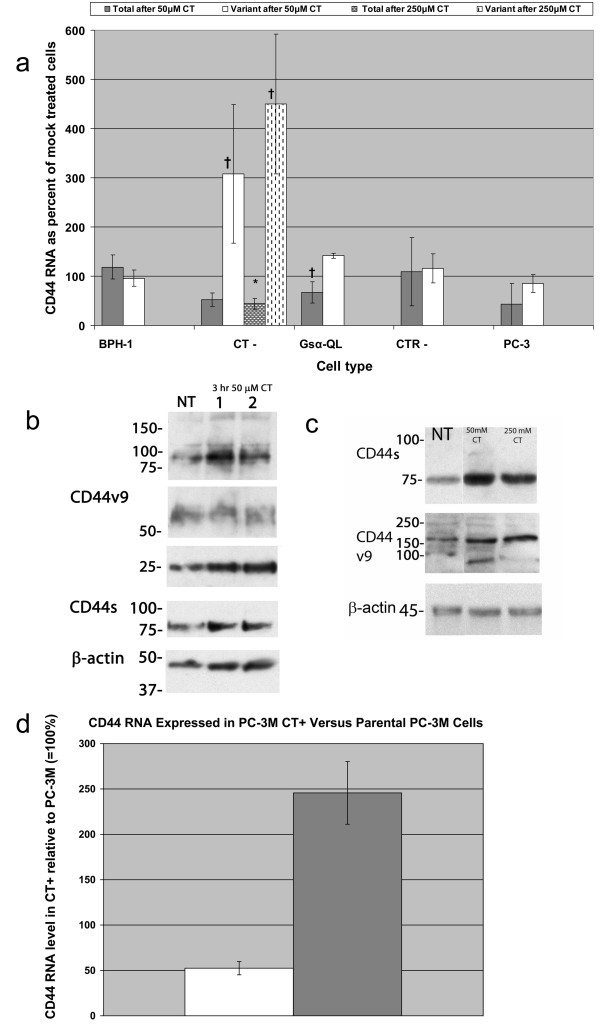
**1a**. RNA response to exogenous CT at 48 h is calcitonin (CT) receptor-dependent. CD44 total and variant RNA are quantified by triplicate TaqMan RT-PCR experiments. In benign BPH-1 cells, no response is observed to exogenous calcitonin (CT). CT-cells, a PC-3M derivative that lack endogenous CT but have CT receptor, showed a decrease in CD44 total RNA but an increase in variant RNA. The G_s_α-QL cell derivative showed the same trend. In CT-receptor-negative CTR- and PC-3 cells, there is no significant response to CT. Error bars are standard deviation. *p = 0.01; ^† ^p = 0.05 with respect to mock treated controls. **1b**. Western blot analysis. Exogenous calcitonin given to the CT-cells for 3 h stimulates expression of CD44s at 75 kD (top), and CD44v at 180 kD and its cleavage products below 97 kD. Comparison is shown to cells with no treatment (NT). β-actin analysis control confirms equal protein loading (bottom). **1c**. This stimulatory effect persists at 48 h and at doses of 50 mM or 250 mM exogenous calcitonin. Also, a time-dependent reduction in protein was observed after cycloheximide treatment, suggesting that CT effect on CD44 variant requires *de novo *protein synthesis and does not result from protein stabilization. **1d**. CT+ cells, endogenously expressing CT, are derived from PC-3M cells and have increased RNA for CD44v (p = 0.02) but a decrease in CD44 total (p = 0.008).

In the PC-3M-derived CT-cells, a 50 or 250 μM CT dose after 48 h had little effect on the total amount of CD44 RNA, but the CD44v was tripled (Fig. 1a). Although different binding affinities of primer/probe sets preclude determining CD44v as a percent of CD44 total, the *relative *percent of CD44v RNA can be calculated by the 2(-ΔΔC_T_) method, as increasing fivefold after 50 μM CT. The same response, but less marked, was seen in G_s_α-QL cells, at 50 and 250 μM doses. In CTR-cells and PC-3 cells – both lacking CTR – exogenous CT had little effect. Similarly, BPH-1 cells responded to CT with very slight stimulatory effect on CD44v, and no effect on CD44 total. At the protein level, however, the CT-cells treated with CT showed increases in both total and variant CD44 after just 3 h (Fig. 1b) and at 48 h (Fig. 1c). The stimulation of CD44v protein was attenuated by cycloheximide up to 9 h after CT (data not shown). This suggests that *de novo *protein synthesis is required and that upregulation of CD44v is not simply a result of protein stabilization.

In CT+ cells, CD44v mRNA doubled compared to PC-3M while CD44 total expression was cut in half (Fig. 1d). This suggests that endogenous CT exerts an increase on CD44 variant similar to exogenous CT.

### Protein kinase A and MAP kinase pathways and their interaction with calcitonin

**Figure 2:**
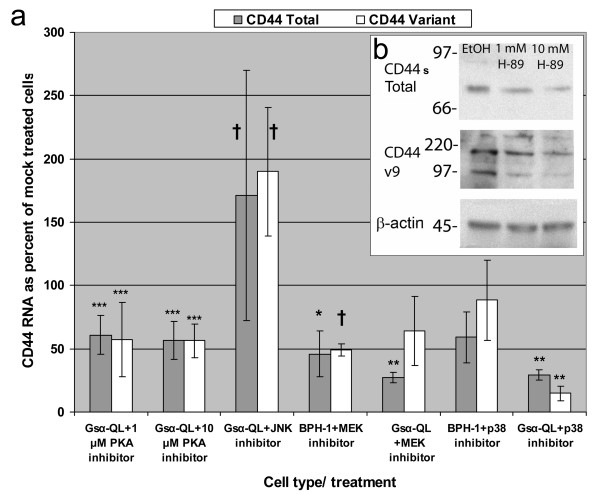
**2a**. RNA response to molecular inhibitors. CD44 total and variant RNA in triplicate TaqMan RT-PCR experiments. In subconfluent G_s_α-QL prostate cancer cells, significant decreases of about 50% or more were observed after protein kinase A (PKA) inhibitor H-89, or inhibitors of downstream signaling pathways MEK or p38 kinase, but not JNK inhibitor. Error bars are standard deviation. *p = 0.01; **p = 0.001; ^†^p = 0.0002; ***p = 0.0001 with respect to mock treated controls. **2b**. Western blot analysis. Protein kinase A inhibitor exerts a dose-dependent decrease on CD44s and CD44v compared with EtOH control in G_s_α-QL cells.

G_s_α-QL cells have high basal levels of CD44v7-10; for this reason, these cells were used to examine the effects of protein kinase A (PKA) and MAPK pathway inhibitors. PKA inhibitor lowered CD44 total and CD44v mRNA (Fig. 2a) and dose-dependently decreased protein for both (Fig. 2b). Downstream to PKA, inhibition of MEK significantly decreased CD44 total (p = 0.001) and non-significantly decreased CD44v. In contrast, inhibition of JNK had no significant effects. p38 inhibitor led to a larger, significant decrease in CD44 variant and a smaller significant decrease in CD44 total (Fig. 2a). MEK and JNK inhibitors were also tested in PC-3 cells and had no effect (data not shown). MEK inhibitor was also tested in BPH-1 cells, in which it reduced CD44 total and variant RNA.

**Figure 3:**
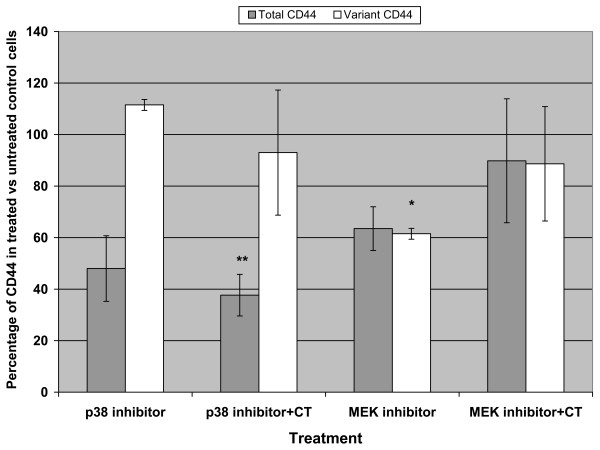
In CT-cells, the blockage of p38 kinase counteracts exogenous calcitonin (CT) stimulatory effect on CD44 variant RNA expression. Triplicate TaqMan RT-PCR experiments. While p38 blockade does not affect the CT-induced decrease in CD44 total, it does abrogate the expected tripling (Fig. 1) in CD44 variant. Conversely, MEK inhibitor moderately reduced total and variant CD44, and this effect was not counteracted by CT, suggesting CT does not act downstream of MEK. *p = 0.02; **p = 0.006 with respect to mock treated controls.

To examine the dependence of CT effects on MAPK pathways, the CT-cells were pretreated with p38 inhibitor 4 h prior to administration of CT. Results were similar to p38 inhibitor alone: more than 50% decrease in CD44 total but none in CD44v (Fig. 3). This lack of CD44v suppression contrasts with p38 inhibitors marked CD44v suppression in G_s_α-QL cells (Fig. 2a), which have far higher CD44v[4]. This suggests that CT mediated splicing is through p38 kinase. In further support of this, the expected CT induced tripling of CD44v mRNA in CT-cells (Fig. 1a) was prevented by p38 inhibitor pretreatment. Pretreatment with MEK inhibitor before CT also blunted the expected rise in CD44 variant mRNA seen in Fig. 1a, and JNK inhibitor pretreatment had no effect (data not shown).

## Discussion and conclusion

**Figure 4:**
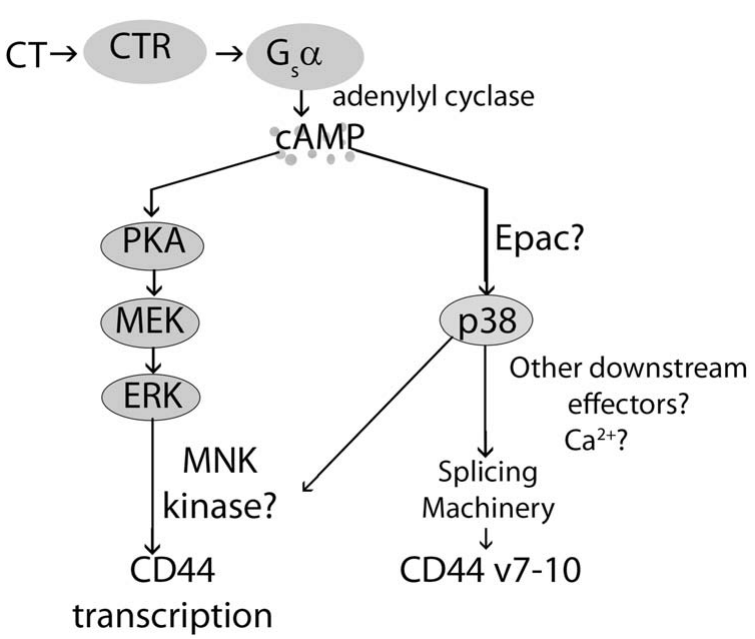
**Proposed effects of calcitonin and MAP kinase pathways on CD44 expression in androgen-independent prostate cancer**. Calcitonin (CT) binds to its receptor (CTR), which is coupled to the G_s_α transduction protein. G_s_α activity, mediated through cAMP, activates protein kinase A (PKA) [19,25]. PKA activates the MAPK kinase (MEK)-extracellular regulated kinase (ERK) pathway, that facilitates CD44 transcription. CT also induces splicing of CD44 to include v7-10, dependent on p38 but not on PKA. p38 may be induced by Exchange protein activated by cAMP (Epac). p38 could affect splicing machinery directly, through other downstream effectors, or by causing release of intracellular Ca^2+^.

Here, we demonstrate that calcitonin (CT) causes CT receptor-dependent increases in CD44 alternate splicing in prostate cancer (PC), apparently mediated through p38 kinase. Furthermore, transcription but not splicing appears to require the MEK/ERK (MAPK) pathway. Proposed interactions are shown (Fig. 4).

Paracrine CT is among several growth factors that interact with CD44[22]. In our prior *in vitro *studies up to 100 nM exogenous CT[6], or CT originating endogenously (in a PC-3 derivative called CT+ [4]), increased CD44v7-10 expression at the mRNA and protein levels. This was also observed in LnCaP, PC-3, and PC-3M derived cells; however, we had not examined total CD44 previously. Here, we used CT-minus (CT-) cells, an androgen-independent PC-3M derivative, to exclude all endogenous CT influence, so any effects would be attributable solely to exogenous CT. In CT-cells, the aberrant splice product is CD44v7-10[6]. This action occurred also in G_s_α-QL cells, which are CTR+. Finally, CT+ cells showed an increase in CD44v compared with PC-3M cells. Supporting the view that this stimulation was CT-receptor mediated and not nonspecific, administering CT to CT-receptor negative PC-3 or CTR-cells did not have this effect. In further support of this interpretation, with benign BPH-1 cells, which are also negative for CT and CTR (personal communication, GV Shah), exogenous CT exerted no effect.

It is a novel observation that CT increases CD44v mRNA and protein as early as 3 h in cells that are CTR+, and has little effect on CD44 total. A response to CT should occur in the first several hours (personal communication, GV Shah), and indeed these increases were evident in CD44 protein levels at 3 h and 48 h.

Since the MEK/ERK pathway and the two stress-activated MAPK pathways are implicated in androgen-independent prostate cancer growth[32], we tested all three for modulation of CD44. We had found that CD44v7-10 protein was overexpressed in xenografts of G_s_α-QL compared to PC-3M[3]; and *in vitro*, pharmacologic stimulation of G_s_α or adenylyl cyclase raised CD44v7-10[6]. Using a PKA inhibitor, we found reduced total and CD44v7-10 mRNA, suggesting involvement of MEK pathway. Over half of PC cases have activated MEK-ERK signaling, shown by immunohistochemistry for p44/ERK1 and p42/ERK2[33].

To assess MAPK inhibitor effects, we chose G_s_α-QL cells, derived from metastasizing PC-3M cells that stably express *gsp *mutant, constitutively active G_s_α [17,19] because they have high baseline CD44v7-10[3,6]. We found MEK inhibitor caused similar percent decreases in CD44 total and CD44 variant, implicating MEK in transcription if not CD44v splicing. Similar effects have been noted with ras oncoprotein, which acts on the MEK-ERK pathway[24]. Ras activation can induce CD44 promoter activity in fibroblasts, as shown using transient cotransfection of c-ras expression constructs and CD44 promoter reporter gene constructs[7]. Leakiness of splice control is proposed to lead to increased CD44v[7]. In activated T-lymphocytes during the immune response, mutant *ras *stimulation of MEK-ERK pathway increases CD44 total mRNA and triggers inclusion of CD44v exons in the mature RNA[24]; from our experiments, MEK seems to be active at least in CD44 transcription in G_s_α-QL cells.

Some studies have suggested a positive feedback loop coupling MEK/ERK pathway and CD44v splicing. Activation of *ras *oncogene in rat fibroblasts[7] and HeLa cervical cancer[23] and of its effector, the MAP kinase pathway in T-cells[24] both upregulate CD44v splicing. CD44 variants, in turn, serve as coreceptors for growth factor receptors that activate ras[23] or form complexes with receptor tyrosine kinases such as c-met[8,21] to mediate cell signaling. Moreover, CD44v6 promotes T-cell proliferation by persistently activating MAP kinases[20], and CD44v8-10 causes apoptosis resistance in small cell lung cancer by activating Rho-stimulated focal adhesion kinase (FAK)[34].

We examined p38 kinase in benign and PC cells. In BPH-1, CD44 total RNA decreased with p38 kinase inhibition but variant form was unchanged. Since benign prostate lacks the aberrant splicing leading to CD44v7-10[3], but CD44v3-10 expression is present[35], the latter may be the form detected in benign cells. In G_s_α-QL cells, a more marked effect on total and variant CD44 was seen. However, p38 may have CT-independent actions. Similar to our current and previous[6] findings with CD44 and G_s_α, p38 and MEK (but not JNK) were responsive to G-protein-coupled P2Y purinoceptor agonist ATP in PC-3 cells (CTR-negative), and these 2 pathways were required for invasion[30]. p38 has recently been recognized as a cell proliferation and survival factor in PC[36] partly by regulating IL-6 secretion[32]. Taken together with our findings about the MEK/ERK role in CD44 transcription, this could reflect convergence of the ERK1/2 and p38 systems in activating the MNK1 kinase, which enhances transcription of certain targets[37], suggesting a common final pathway that stimulates CD44 expression in PC.

We tested possible JNK pathway effects on CD44, not previously examined in the literature. JNK appears mainly important in PC apoptosis[29,38] and promoting chemotherapy susceptibility. JNK inhibitor slightly decreased CD44 total protein and did not change CD44v mRNA or protein in G_s_α-QL cells. Our data suggest that JNK is a minor influence on CD44 expression.

Inhibition of p38 and MEK pathways affected CD44 in G_s_α-QL cells. To investigate whether either might mediate CT's effects on CD44, we administered CT to CT-cells after blocking either one of these pathways. CT-cells have low baseline CD44v[6], and p38 inhibitor did not suppress the CD44v, but it blocked the expected stimulation of CD44v by CT, suggesting that p38 mediates CT-stimulated alternative splicing. The marked CD44v suppression seen in G_s_α-QL cells, which have endogenous CT and high baseline CD44v, adds support for this interpretation. It is tempting to speculate that CT signaling, raising cAMP, may act through the effector, "exchange factor directly activated by cAMP" (Epac, Fig. 4). Epac has been shown to activate p38 kinase and mobilize intracellular calcium in neurons[39]. This PKA-independent mechanism would explain why PKA affected primarily CD44 transcription, yet p38 showed evidence of an additional effect on splicing.

To our knowledge, this is the first report in PC of how interactions between CT, and MAP kinase pathways, dysregulate the expression and splicing of the CD44 molecule. CD44 variant isoforms, probably through alterations in multimerization[12] and ligand binding[4], allow prostate cancer invasion[3,6]. This knowledge may find application in targeting the aberrant splicing of CD44 in PC by gene therapy, molecular inhibitor therapy, or for sensitization to radiotherapy.

## Abbreviations

CD44: cell determinant 44; CT: calcitonin; CTR: calcitonin receptor; ERK: extracellular signal-regulated kinase; JNK: Jun N-terminal kinase; MAPK: mitogen-activated protein kinase; PC: prostate cancer; PKA: protein kinase A.

## Competing interests

The authors declare that they have no competing interests.

## Authors' contributions

EWR and EAT conducted experiments; EAT performed statistical analysis; KY did some western blot analyses; KAI conceived of the study and wrote much of the manuscript.

## Pre-publication history

The pre-publication history for this paper can be accessed here:


